# Therapeutics

**Published:** 1876-06

**Authors:** 


### III. Therapeutics.

1. *Treatment of Rheumatic Fever with Salicylic Acid.* (Ed. *Times & Gaz.*, 1876.)

We extract the following details from a paper by Staff-Surgeon Stricker, in the *Berliner Klinische Wochenschrift* of January 3, 1876. For several months all the cases of acute rheumatism in which the local symptoms were strongly marked (fourteen in all) were treated with salicylic acid. The preparation used was, however, not the ordinary impure commercial acid, but one that by repeated crystallization had been rendered almost perfectly pure. Thus prepared it consists of shining white needles, which have no smell, and dissolve completely in water and alcohol so as to form a clear solution. This pure acid can be given internally in considerable doses without any of those unpleasant results which have followed the use of the commercial acid, which probably owes its caustic properties to the presence of other substances—for instance, carbolic acid. The pure acid only excites some dryness in the mucous membrane of the mouth and pharynx, followed by an increased secretion from their surfaces. This inconvenience can, however, be obviated by giving the acid in half-gramme or gramme doses every hour, in the form of powder, and enclosed in a capsule ; and in the treatment of rheumatic fever the administration is continued until the joints which were previously affected can be moved without pain. To quote Dr. Stricker's words, "All the patients thus treated were not only relieved of their fever, but also of the local symptoms—*i. e.*, the swelling, redness, and especially the painfulness of their joints—within forty-eight hours ; most of them even within a much shorter period." The largest quantity of pure salicylic acid which was found necessary to produce this effect was fifteen grammes, and the smallest five grammes ; but that even larger quantities can be taken internally without injuring the digestive apparatus is proved by the fact that one patient actually took twenty-two grammes in the course of twelve hours through an excess of zeal on his own part ; but, nevertheless, his tongue became clean and his appetite returned in the course of this vigorous drugging. As far as Dr. Stricker's observations go, the more acute the case the better the action of the acid. He finds it best to begin the treatment in the morning, for then its effects are generally so decided by the evening that it is unnecessary to disturb the patient's rest to give him his medicine. The general phenomena which were observed to follow large doses of the acid were copious perspiration, ringing in the ears, and slight deafness, and in two cases the patients became more than usually lively. Dr. Stricker does not pretend to express any opinion at present on the effect exerted by the acid on the cardiac complications of rheumatic fever. Most of his cases either had old valvular disease or else were suffering from a recent endocarditis at the time of their admission. The details of five cases are appended to Dr. Stricker's paper, and the temperature-sheets are given in graphic form in four of them, and we can only say that they confirm in a most striking manner what has been above stated as to the value of the salicylic treatment.

A further testimony in the same direction is that of Dr. L. Riess (*Berl. Klin. Wochenschrift*, December 20, 1875, "On the Internal Use of Salicylic Acid"). He speaks of the results obtained in acute rheumatism as unexpectedly favorable, and points out that the action of the salicylic acid must be more than purely symptomatic, since in some cases a single dose not only permanently reduced the temperature, but also was followed by general improvement in the patient's condition. We again remind our readers that the acid used must be CHEMICALLY PURE.

2. *The Indications for Quinine in Surgical Practice.* VERNEUIL. (*Gaz. Méd., Med. Times & Gaz.*, 1876.)

M. Verneuil states that quinine may be beneficially employed in treating ataxic, neuropathic and septicæmic complications. 1. It is not necessary for the ataxic phenomena to be of a true intermittent type, for quinine exercises its regulative action when no palustral element exists. 2. It is especially valuable in combating neuropathic phenomena, and nowhere can its influence be better appreciated than in affections of the eye. M. Verneuil is in the habit of prescribing it after operations on this organ, and he has frequently obtained most remarkable results. 3. In septicæmic accidents the salutary action of quinine is beyond all doubt, and given in large doses it still constitutes the best agent we have to oppose to purulent infection. 4. As an antiseptic it appears to act in two modes—modifying and diminishing the formation of pus, and operating as a direct antiputrefactive—so that it is advantageously employed as a topical antiseptic. 5. Its surgical employment is especially indicated for women and children, who are more disposed than men and adults to ataxic and neuropathic accidents.

3. *Treatment of Epistaxis by the Internal Administration of Ergot.* (*British Medical Journal.*)

Dr. George St. George, of Lisbon, observes that the treatment of epistaxis is often attended with great difficulty, especially in persons enfeebled by age. He has found ergot of use in cases where liquor ferri perchloridi, plugging, and other remedies had been tried without avail. The following is one of the cases he records. A weak anæmic woman, aged 55, had been suffering for three days from repeated and violent attacks of hæmorrhage from the nose, which had increased in both frequency and violence during the last twenty-four hours. The nostrils were first plugged with lint and cold water. Dr. St. George then tried plugging with lint dipped in liquor ferri perchloridi, but without avail, as the hæmorrhage continued. He then ordered her a mixture, each dose to contain fifteen minims, of liquid extract of ergot every quarter of an hour until the hæmorrhage ceased, and then to be continued every four hours for a day or two. In an hour and a half the bleeding had entirely ceased and never returned. He gives the details of two other cases successfully treated by the same means.

4. *Action of Jaborandin, or Chlorhydrate of Pilocarpine.* (*Centralblatt, f. d. Chirurgie*, No. 52, 1875.)

The alkaloid obtained from the summitates jaborandi (pilocarpus pinnatus), termed jaborandin, or, better, pilocarpin, has a more uniform action than the leaves themselves, and, according to Siredey, acts somewhat differently from them. The doses recommended by Dr. Dumas in his thesis are from half a grain to two grains, from which no injurious influence need be anticipated. The action on the salivary glands is the most marked, and commenced very soon after its administration, so that in the course of five hours a rapidly increasing, and then slowly diminishing, salivation took place, especially from the submaxillary glands. The total excretion of saliva in this period amounted to 755 grammes (11,657 grains, more than 1½ lbs. avoir.). Copious sweating occurred one hour after the commencement of the salivation, which slowly diminished in the course of four hours. Biliary vomiting usually occurred, the urinary secretion was greatly diminished, the pul-e was slowed, the temperature lowered, the pupil dilated. No therapeutic value has as yet been assigned to it.

5. *Therapeutic Value of Picrotoxine.* (*Gazette Médicale de Paris*, No. 51, 1875.)

According to recent researches, *picrotoxine* exerts a special action on the medulla spinalis and oblongata. M. Gubler has hence been led to employ it in a case of labio-glosso-pharyngeal disease, and obtained well-marked improvement after a few days. The patient, in fact, who, on admission, was only able to take liquid food, consumed the usual diet of the hospital; the salivation had disappeared, and speech had become more easy. Picrotoxine has been administered in doses of one milligramme (.01544 grain). At the point where the injections were made, small, hard, indolent tubercles, resembling syphilitic gummata, appeared, which slowly disappeared. This hyperplastic action of picrotoxine on the cellular tissue is, as M. Gubler observes, interesting to notice. M. Dujardin-Beaumetz has tried the effect of picrotoxine in epilepsy, beginning with smaller doses than the above, and attaining ultimately three milligrammes per diem. Here, too, prompt amelioration took place in the symptoms. The fits, which in the first instance occurred every day, only took place every second day, then became more and more rare, till they ultimately disappeared altogether. The patient quitted the hospital after two months, and had not again been seen, though he promised to return should the fits recur. This result does not, of course, demonstrate that the epilepsy was cured, but simply that an attack had been staved off for two months; but it suggests that further trials should be made with it. No results have been obtained from the employment of picrotoxine in paralysis agitans.

6. *Electricity, its Therapeutic Applications.* (*The Practitioner*, 1876.)

The electric force in all forms is a disturbing element, and is therefore unsuited to the conditions in which *rest* is the indication.

It may be assumed that this agent is inapplicable in all cases of recent or acute inflammation. After the subsidence of the acute symptoms, however, this agent may be beneficial. The same is true of friction and vibration. The appreciation of this principle will save some disappointments from the misapplication of the remedy.

Headache from acute inflammation or acute congestion will, according to this principle, be aggravated by electricity. A headache from inanition or anæmia will be benefited, and sometimes instantly removed by electricity. A sympathetic headache will be removed by this agent when the sympathetic cause is removed.

A dyspeptic headache will generally prove intractable, because the gastric derangement will defy removal by this agency.

On the other hand, a headache dependent upon defective innervation in the sympathetic or ganglionic system, will disappear by the electric stimulation of the sympathetic gangalia. In this case it is not necessary to include the brain within the scope of the current.

In the chest "a stitch in the side," or any form of neuralgia is generally benefited, while a true pleurisy is made worse. In doubtful cases, therefore, the behavior of the agent may be made a means of diagnosis.

In the abdomen, constipation from sluggish muscular action and deficient intestinal secretion is benefited by this agent, while the same symptoms resulting from inflammatory action are only made more intense. The intestinal muscles paralyzed by inflammatory congestion are still more congested by the electric stimulus.

In the loins, the condition of lumbago is favorable for the beneficial action of this agent, while a deep-seated inflammation is unfavorable.

In the pelvis, especially in the female, the organs are benefited or injured, according as we comply with this principle, or neglect or misunderstand it.

A congestion dependent upon local muscular passiveness in the walls of the vessels ought to be benefited, while a true acute or recent inflammation will be aggravated. The practical appreciation of this agent should, upon this basis, be an aid to diagnosis.

Sciatica follows the rule of lumbago, while an inflammation of a nerve presents a condition unfavorable for the application of electricity.

An exception may be made in the apparent effects of a powerful galvanic current, which is capable of producing a benumbing effect, even in an inflammatory condition. In this case, the apparent benefit is temporary, the pain soon becoming as bad as before.

7. *The Action of Cold upon Milk and its Products.* TISSERAND. (*L'Union Méd.*, No. 18.)

Fresh cows' milk, submitted to different temperatures, immediately or soon after its withdrawal, presents the following peculiarities, if kept for 24 or 36 hours at the initial temperature (between 0° and 36° C.):

1. The rise of the cream is rapid in proportion as the temperature to which it is exposed approaches the zero point (C.)

2. The volume of cream obtained is greater, according as the milk is subjected to a lower temperature.

3. The butter yielded is more considerable, according as the temperature is reduced.

4. Finally, skim milk, butter and cheese are of better quality in the latter case.

Pasteur's experiments on the origin, alterations and favorable or unfavorable media of ferments, have an application here. It is probable that the energetic cooling arrests the evolution of the living organisms which constitute ferments, and prevents the alterations due to their presence. This treatment of milk produces effects analogous to those observed in the manufacture and preservation of ices and Vienna Beer, which is so remarkable for its quality.

These facts, at any rate, prove how erroneous are the commonly received ideas upon this subject, viz., that milk intended to furnish cream should be kept at a temperature of 12° or 13° C., and not lower.

